# Special Issue: “Functionalized Nanomaterials and Structures for Biomedical Applications”

**DOI:** 10.3390/ma16247521

**Published:** 2023-12-05

**Authors:** Paul Cătălin Balaure

**Affiliations:** “C.D. Nenițescu” Department of Organic Chemistry, Faculty of Chemical Engineering and Biotechnologies, University Politehnica of Bucharest, 011061 Bucharest, Romania; paul.balaure@upb.ro

Nanoscale drug formulations are under wider and wider investigation due to their multiple unique advantages, such as stealth properties which avoid opsonization in the blood stream; specific ligand guided drug delivery to injured cells and tissues; on-demand release in sharp response to a series of endogenous and exogenous stimuli, allowing for predetermined programmed delivery to specific loci in the body and possible timing with circadian rhythms; increased cellular uptake due to their small dimensions and the possibility of intracellular delivery of the therapeutic cargo, especially when functionalized with cell penetrating agents; increased stability in biological fluids; augmented bioavailability; and tailored pharmacokinetics and pharmacodynamics. Other cutting-edge applications of nanomaterials and nanostructures in the biomedical field are theragnostic and personalized medicine approaches in cancer diagnosis, monitoring, and therapy, as well as the prevention, control and treatment of recurrent infections caused by pathogenic biofilms using bioactive nanocoatings that are able to prevent bacterial adhesion and the colonization of surfaces or to dismantle already-settled biofilms. Multidrug resistance in bacteria, a widespread life-threatening condition, can also be addressed by means of smart functionalized nanocarriers. The versatility and plethora of applications lay in the structural and morphological diversity of nanomaterials ranging from simple inorganic compounds to more complex organic materials based on lipids, polymers, block-copolymers, hybrid materials and supramolecular assemblies of tunable morphology.

The first volume of this Special Issue dedicated to biomedical applications of functionalized nanomaterials comprises eleven articles, including nine research papers and two reviews. G. Tejada, D. Leonardi, et al. [1] developed a buccal film based on natural polymers for simultaneous delivery of miconazole nitrate (MN; an antifungal) and lidocaine hydrochloride (LDCH; an anesthetic) in the topical treatment of oropharyngeal candidiasis. They prepared chitosan (CHI)-alginate (ALG) (50–50%) microparticles loaded with MN and then dispersed these microparticles in hydroxypropyl methylcellulose-gelatin-based films containing LDCH. The developed films showed proper characteristics in terms of adhesiveness, thickness, tensile strength, and elongation at break, presenting a fast release of LDCH and a controlled release profile for MN. B. Mubaiwa, E.M. Antunes, D.R. Beukes, et al. [2] presented an interesting in vitro study concerning the improved intracellular delivery of cytotoxic pyrroloiminoquinone metabolites extracted from a South African Latrunculid sponge using a platform based on green-synthesized gold nanoparticles. The aqueous extract of a brown seaweed, *Sargassum Incisifolium*, containing polysaccharides and polyphenols, was employed as a reducing agent in the preparation of gold nanoparticles (sAuNPs). Next, the cytotoxic metabolites were adsorbed onto sAuNPs and the resulting nanoconjugates were evaluated with respect to cytotoxic activity against the breast cancer MCF-7 cell line together with the metabolites alone, the known chemotherapeutic agent doxorubicin as a control and the corresponding gold conjugate of the latter. Both doxorubicin and alkaloid loaded sAuNPs showed high (80–85%) cancer cell penetration while significantly retaining the cytotoxic activity, although this was slightly lower than that in the pyrroloiminoquinone metabolites or antitumor drug alone. An enhanced and more selective antitumor effect is expected to be reached using these sAuNP–cytotoxic metabolite conjugates via the EPR effect especially when used in conjunction with photothermal therapy and theragnostic agents. However, further studies are required to assess the influence of the pH, which is acidic in tumors on the desorption of the alkaloids from the delivery nanoplatform as well as the cytotoxic activity against normal cell lines, before proceeding to in vivo preclinical studies. Other papers deal with various syntheses of nano- and micro-sized particulate systems for biopharmaceutical applications [3,4,5,6,7]. V. Mikelashvili, L. Almasy, et al. [3] presented the characterization of citric acid-capped magnetite nanoparticles synthesized using an electrohydraulic discharge treatment. They used the well-known co-precipitation method to prepare bare Fe_3_O_4_ nanoparticles which were further processed by electrohydraulic discharges with a high discharge current (several tens of amperes) in a water medium using a pulsed direct current, prior to surface modification. The electrohydraulic treatment resulted in improved properties of the prepared nanoparticles in terms of higher saturation magnetization, higher citric acid coverage and better particle dispersion. R. Rajamoham, S.-K. Kim, Y.R. Lee, et al. [4] developed a one pot synthesis of copper oxide nanoparticles using an eco-friendly reducing agent, namely apple peel extract, and aqueous copper nitrate dihydrate as the copper source under microwave irradiation. The antibiofilm activity of the prepared CuO nanoparticles was tested against clinically relevant pathogens *Escherichia coli* and *Staphylococcus aureus*. At a nanoparticle concentration of 25 μg/mL, reductions with respect to untreated controls of biofilm mean thickness and biomass of 85% and 65%, respectively, were evidenced for both biofilms through confocal scanning laser microscopy analysis using COMSTAT software. M. Valentin, B. Vertruyen, et al. [5] reported the successful preparation of spherical macroporous mannitol (M) granules with diameters of about 2–3 μm via a spray drying technique using polystyrene (PS) beads as a sacrificial template and an FDA-approved ethyl acetate solvent as an etching agent. The influence of the M/PS mass ratio on the etching efficiency reflected in the material porosity was studied and the authors also investigated how the homogeneity of the porosity distribution was affected by upscaling the synthesis process from lab-scale to pilot-scale conditions. Given the dimensions of the fabricated mannitol granules, they can be used as a porous fine excipient for efficient pulmonary drug delivery. In an interesting study carried out by S.K. Nagaraja, S. Nayaka, et al. [6], silver nanoparticles were biosynthesized using the phytoconstituents of an aqueous extract of a *Leonotis nepetifolia* flower bud as a reducing, capping and stabilizing agent. Using the MTT assay, spherical biosynthesized LnFb-AgNPs with a mean diameter of 24.5 nm were shown to exhibit cytotoxic activity against PANC-1 (pancreatic ductal adenocarcinoma) cells with an IC_50_ value of 35.84 μg/mL. Moreover, AgNPs capped with *L. nepetifolia* flower bud extract induced significant early (10.71% of the cell population) and late (38.3% of the cell population) apoptosis. When testing their antimicrobial activity with the agar well diffusion method, the LnFb-AgNPs showed maximum growth inhibition zones against *Pseudomonas aeruginosa*, *S. aureus* and *Candida glabrata*. The antioxidant activity of the LnFb-AgNPs was stronger than that of the extract itself as determined with the DPPH assay. All these remarkable characteristics recommend the silver nanoparticles produced via *L. nepetifolia*-mediated phytosynthesis for further in vivo studies. Another green synthetic approach was successfully applied by Dhatwalia et al. [7] to produce ZnO nanoparticles using an aqueous extract of *Rubus* *ellipticus* fruits. The prepared ZnO nanoparticles showed antioxidant, antibacterial, antifungal and anticancer activities in vitro. In terms of MIC, the best antimicrobial activities were observed against the Gram-positive bacterium *Bacillus subtilis* (31.2 µg/mL) and the phytopathogenic ascomycete fungus *Rosellinia necatrix*, respectively. The anticancer activity assessed with the MTT assay revealed a percentage of 52.41% of lung cancer A549 cell deaths at a ZnONP concentration of 200 µg/mL, whereas the IC_50_ value was 158.1 ± 1.14 µg/mL. The ZnO nanoparticles also showed effective photocatalytic activity; they degraded 17.5% of the dye methylene blue at a concentration of 10 ppm in dye-tainted wastewater within 1 h, reaching a final degradation percent of 72.7% after 3 h, which suggests the application of these nanoparticles in water pollutant removal. M.M. Eshaghi, A. Rahdar, A.M. Diez-Pascual, et al. [8] developed novel pH-responsive carboxymethylcellulose (CMC)-based hydrogel encapsulating core–shell Fe_3_O_4_@SiO_2_ nanoparticles for controlled delivery of the plant flavonol quercetin (QC). In order to achieve extended drug release, the nanocomposite hydrogel was encapsulated in a double-layer w/o/w emulsion. Drug release kinetic studies showed a pH-dependent release profile. In neutral medium, most of the payload was retained within the prepared nanoemulsion, while in acidic medium protonation of the carboxyl groups followed by hydrogen bond dissociation resulted in increased QC release. When tested with the MTT assay and flow cytometry against the A549 cancer cell line, the QC-loaded nanoemulsion showed higher capacities to kill and induce apoptosis of cancer cells than free QC. Furthermore, the MTT assay against the normal fibroblast L929 cell line demonstrated low cytotoxicity compared with the free drug, suggesting that the pH-responsive composite nanoemulsion would significantly reduce the serious side effects of cytostatic therapy. To mitigate the severe cardiotoxicity of tyrosinase inhibitors (TKIs), a new generation of potent antiproliferative and anti-growth agents, Al-Thani et al. [9] loaded the smart triblock copolymer PLGA-PEG-PLGA, in which the middle hydrophilic homopolymer segment is flanked by two hydrophobic polymer segments, with the TKI Ponatinib. They transplanted the human myelogenous leukemia cell line K562 into zebra fish embryos and used this xenograft as a suited in vivo animal model to assess the toxicity of both genuine and Ponatinib-loaded polymeric nanoparticles at various concentrations in terms of survival rate and an analysis of their cardiovascular structure and function. At a concentration of 0.001 mg/mL, the Ponatinib-loaded PLGA-PEG-PLGA nanoparticles were shown to be non-toxic/non-cardio-toxic. A.M. Diaz-Pascual provided a nice review [10] tackling the most recent achievements in the field of engineered nanostructures in terms of design and functionalization along with important applications in cancer treatment, tissue engineering, drug/gene delivery and medical implants. H. Ashraf et al. [11] highlighted the most relevant progress in investigating the role of selenium nanoparticles in the regulation of altered microbiota and in the treatment of neurological diseases.

The interest raised by the proposed research topic prompted us to open a second volume of “Functionalized Nanomaterials and Structures for Biomedical Applications”. We cordially invite outstanding scholars worldwide to contribute to this open access Special Issue, thereby increasing the visibility of their remarkable research.
